# Cryo-electron microscopy of chromatin biology

**DOI:** 10.1107/S2059798317004430

**Published:** 2017-04-20

**Authors:** Marcus D. Wilson, Alessandro Costa

**Affiliations:** a Macromolecular Machines Laboratory, The Francis Crick Institute, 1 Midland Road, London NW1 1AT, UK

**Keywords:** nucleosome, chromatin, cryo-EM, cryo-electron microscopy, histone modification, integrase

## Abstract

The basic unit of chromatin, the nucleosome core particle (NCP), controls how DNA in eukaryotic cells is compacted, replicated and read. Since its discovery, biochemists have sought to understand how this protein–DNA complex can help to control so many diverse tasks. Recent electron-microscopy (EM) studies on NCP-containing assemblies have helped to describe important chromatin transactions at a molecular level. With the implementation of recent technical advances in single-particle EM, our understanding of how nucleosomes are recognized and read looks to take a leap forward. In this review, the authors highlight recent advances in the architectural understanding of chromatin biology elucidated by EM.

## Abstract 2

This review article summarizes recent progress in our understanding of chromatin biology based on single-particle cryo-electron microscopy studies.

## 1. Introduction   

**Figure 1:**
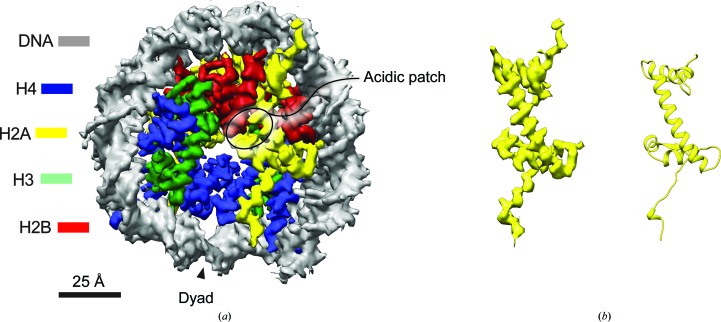
(*a*) NCP structure. The 3.9 Å resolution structure from Chua *et al.* (2016 ▸) (EMD entry 8140) displayed and segmented in *UCSF Chimera* (Pettersen *et al.*, 2004 ▸) to highlight the different histone proteins and DNA. Key NCP features are highlighted. (*b*) The density for H2A is displayed next to the ribbon representation of H2A from Davey *et al.* (2002 ▸) (PDB entry 3lz0).

Each human cell contains over 2 m of DNA that is highly compacted by chromatin into the cell nucleus, which in turn measures only around 50 µm^3^. Structural biology approaches have started to reveal how DNA is compacted and modified in the cell. For example, early electron-microscopy (EM) work confirmed biochemical conclusions that the basic unit of chromatin is the nucleosome, which compacts DNA around a central discoid of tetrameric H3–H4 capped by H2A–H2B dimers on either face (Richmond *et al.*, 1984 ▸; Klug *et al.*, 1980 ▸). The nucleosome core particle (NCP) is roughly 10 nm in width, with 1.65 turns of DNA wrapping around the equator of the coin-shaped particle. The landmark publication of the 2.8 Å resolution structure of the nucleosome (Luger *et al.*, 1997 ▸) revealed the key features of the histone fold and protein–DNA interactions. The near-atomic map was made possible in part by the use of entirely recombinant histones and strong-positioning DNA, reassembled to create a more homogenous population of NCPs than those isolated from cells. Each histone exhibits a characteristic three-helical dumbell shape, with largely unstructured N- and C-terminal tails (Fig. 1 ▸). The DNA contacts the octameric disc, with numerous basic residues that map onto the outer perimeter of the histone core and project into the DNA minor groove, engaging in non-sequence-specific interactions. The solvent-exposed upper and lower faces of the nucleosome form an undulating surface with distinct electrochemical features used for chromatin protein recognition. The histone tails are the major site of post-translational modification (Ng & Cheung, 2016 ▸; Ruthenburg *et al.*, 2007 ▸); they have been described to be in multiple conformations and are likely to be highly flexible (Luger *et al.*, 1997 ▸; Davey *et al.*, 2002 ▸; Hansen *et al.*, 2006 ▸) but can become ordered upon protein binding (Armache *et al.*, 2011 ▸; Arita *et al.*, 2012 ▸). The NCP provides a platform that facilitates the reading and copying of the bound DNA and helps to control the myriad of DNA-related processes in the cell. The relative scarcity of nucleosome structures represents a major obstacle in understanding how nucleosomes are modified, read, unwrapped, removed and deposited. X-ray crystallographic studies have revealed how different histone variants and DNA sequences affect the core NCP (reviewed in McGinty & Tan, 2015 ▸). However, despite recent progress, relatively few X-ray structures of NCP-bound complexes, epigenetically modified NCPs and higher order NCP arrays exist (McGinty & Tan, 2016 ▸).

Complementary structural techniques such as NMR, SAXS and EM have been developed to visualize chromatin components, with some advantages over X-ray crystallography. A number of studies have used methyl-TROSY NMR to help to build structural models of dynamic nucleosome interactions (Eidahl *et al.*, 2013 ▸; Kato *et al.*, 2011 ▸; Zhou *et al.*, 2013 ▸). Lower resolution models of NCP complexes have been extrapolated from SAXS (Pilotto *et al.*, 2015 ▸) and single-particle EM data (Nguyen *et al.*, 2013 ▸; Chaban *et al.*, 2008 ▸; Saravanan *et al.*, 2012 ▸; Tosi *et al.*, 2013 ▸; Yamada *et al.*, 2011 ▸). Indeed, EM has long been used in cell slices and *in vitro* reconstituted chromatin fibres to probe the arrangement of nucleosomes in higher order structures (Scheffer *et al.*, 2011 ▸; Robinson *et al.*, 2006 ▸; Routh *et al.*, 2008 ▸; Finch & Klug, 1976 ▸; Eltsov *et al.*, 2008 ▸). However, with the advent of new imaging technology and computational methods (Fernandez-Leiro & Scheres, 2016 ▸), single-particle cryo-EM can now provide molecular detail on previously intractable complexes. Three factors make structural cryo-EM an attractive technique: (i) preparations require comparatively low amounts of soluble, monodisperse sample; (ii) a certain degree of compositional/conformational heterogeneity, which would generally impair crystallization, can be handled computationally; and (iii) larger macromolecules, such as nucleosome-containing assemblies, are easier to visualize in the electron microscope.

In this review, we will summarize recent advances in determining single-particle EM structures of nucleosome biology assemblies, highlighting the changing role and advantages of EM approaches compared with more conventional structural biology tools.

## 2. Visualizing nucleosomes under the electron microscope   

Despite their relatively small size (∼200 kDa), nucleosomes are highly compact and provide relatively high contrast in cryo-EM owing to increased electron scattering from the wrapped nucleosomal DNA. Despite these advantages, identifying small NCP particles in vitreous ice can be challenging, and most cryo-EM NCP structures to date have been visual­ized with added mass, either from bulky post-transitional modifications (Wilson *et al.*, 2016 ▸) or in complex with large protein assemblies (Maskell *et al.*, 2015 ▸; Yamada *et al.*, 2011 ▸; Xu *et al.*, 2016 ▸). Chua and coworkers circumvented this problem by using a Volta phase plate, allowing increased contrast at low spatial frequencies and improved particle alignment (Chua *et al.*, 2016 ▸). These authors were able to reconstruct a final cryo-EM map with a resolution of 3.9 Å, which agreed well with available crystallographic structures of NCPs (Fig. 1 ▸). By comparing the EM density with the higher resolution published crystal structures, subtle details of the histone tails can be resolved (Chua *et al.*, 2016 ▸; Wilson *et al.*, 2016 ▸; Fig. 1 ▸). Compared with the EM maps, the path and density for the histone H3 and H4 tails are better ordered in crystal structures. However, even from the lower resolution EM maps the N- and C-terminal tails of histone H2A can be observed, suggesting these may be well ordered in isolated particles in vitreous ice (Fig. 1 ▸).

## 3. Using single-particle EM to study NCP interactors   

Only a handful of chromatin-binding protein–NCP structures have been determined by X-ray crystallography (Barbera *et al.*, 2006 ▸; Makde *et al.*, 2010 ▸; Armache *et al.*, 2011 ▸; McGinty *et al.*, 2014 ▸; Girish *et al.*, 2016 ▸; Morgan *et al.*, 2016 ▸; Fang *et al.*, 2016 ▸; Zhou *et al.*, 2015 ▸). All structures to date utilize multiple elements of the surface of the NCP to garner nucleosomal specificity and affinity, engaging in a multivalent manner. Multivalency may be imparted *via* multiple contacts within the same domain. Examples of this type of interaction have been reported for the known crystal structures of Rcc1 and the Sir3 BAH1 domain (Makde *et al.*, 2010 ▸; Arnaudo *et al.*, 2013 ▸; Armache *et al.*, 2011 ▸). Alternatively, cooperative binding to NCPs could be built up through the genetic linkage of different chromatin-binding domains into a single polypeptide or several reader domains within the same protein complex. Indeed, tandem-adjacent histone code-recognition modules have been found in multiple proteins (Ruthenburg *et al.*, 2007 ▸; Ng & Cheung, 2016 ▸; Taverna *et al.*, 2007 ▸). Intriguingly, many nucleosome binders utilize a region of high negative charge, termed the acidic patch, formed between residues in histones H2A and H2B (Fig. 1 ▸). A common arginine anchor motif has been described in all acidic patch interactors to date (McGinty & Tan, 2016 ▸; McGinty *et al.*, 2014 ▸).

Before the ‘resolution revolution’ in cryo-EM, single-particle studies of nucleosome-containing complexes were of limited resolution (>20 Å; Nguyen *et al.*, 2013 ▸; Chaban *et al.*, 2008 ▸; Saravanan *et al.*, 2012 ▸; Tosi *et al.*, 2013 ▸; Yamada *et al.*, 2011 ▸; Chittuluru *et al.*, 2011 ▸). Although only the overall domain organization can be described at such resolutions, these studies have been important in providing a first insight into how large flexible molecular machines remodel nucleosomes on DNA. A series of illustrative EM studies investigated the mechanism of H2A histone variant H2AZ exchange, which is catalysed by the opposing action of INO80 and SWR1 chromatin-remodeller complexes. Both complexes are highly dynamic and in excess of 1 MDa. The EM structures revealed that the complexes have a common architecture, with a large AAA+ ATPase head and an extended flexible tail composed of distinct polypeptides (Watanabe *et al.*, 2015 ▸). Negative-stain EM revealed that INO80 undergoes a conformational change, clamping the NCP between the Arp8-containing tail domain and the ATPase head domain (Saravanan *et al.*, 2012 ▸; Tosi *et al.*, 2013 ▸). In contrast, SWR1 forms far more limited contacts with the NCP, with only one face of the NCP engaged primarily by the catalytic Swr1 subunit (Nguyen *et al.*, 2013 ▸). This observation may explain the different functional activities of INO80 and SWR1: while both can evict and replace H2A variants, only INO80 can slide NCPs along DNA.

The RSC chromatin remodeller promotes nucleosome translocation and contains a preformed cavity to engage the NCP (Asturias *et al.*, 2002 ▸). A cryo-EM study of RSC–NCP showed a clear signal for the histone octamer in the centre of the RSC cavity (Chaban *et al.*, 2008 ▸). Poorer density for the nucleosomal DNA was observed, suggesting that RSC binding leads to partial separation of DNA from the octamer, with looping that allows DNA translocation. A separate study used cryo-EM combined with X-ray crystallography to characterize a DNA-binding portion of the ISW1a remodelling complex (Yamada *et al.*, 2011 ▸). Crystal structures of the ISW1a core lacking the catalytic ATPase domain revealed the asymmetric binding mode to two strands of DNA. The 24 Å resolution cryo-EM structure of a dinucleosome revealed that the DNA-binding portion of ISW1a is well positioned to recognize both the entry and exit of DNA from an NCP and to help to define the spacing between nucleosomes, acting as a direct molecular ruler.

**Figure 2:**
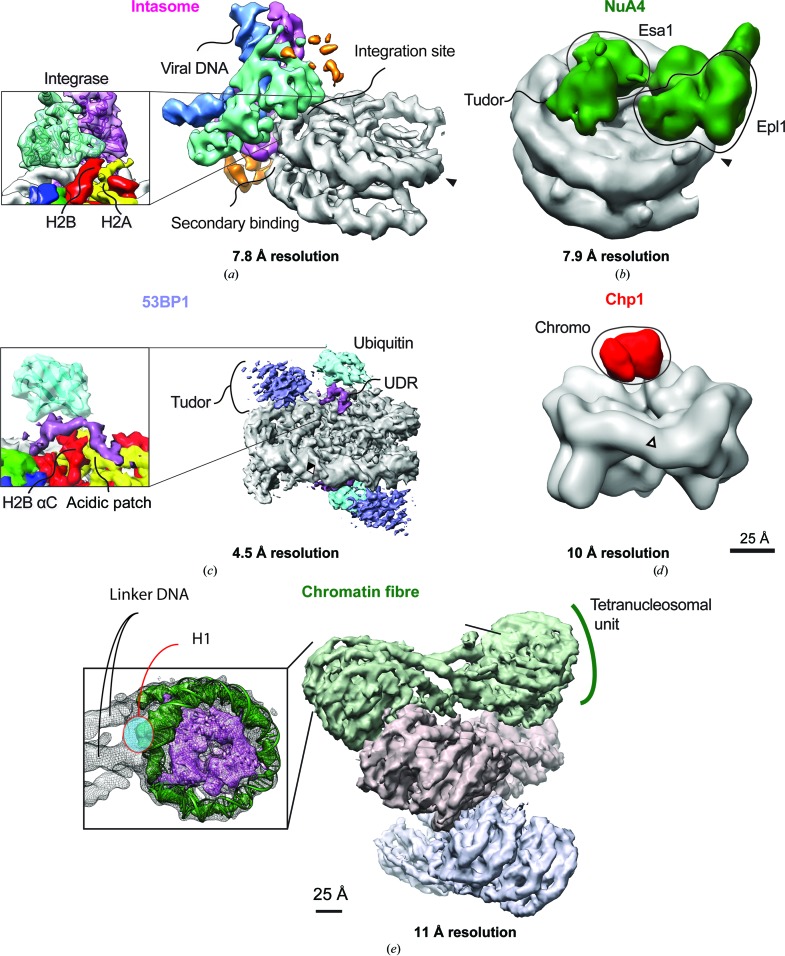
Near-nanometre and sub-nanometre NCP–interactor EM structures. EM density maps were displayed and segmented in *UCSF Chimera*. Key domains commented on in the text are highlighted and the position of the NCP dyad is labelled with an arrow. (*a*) PFV intasome–NCP structure (EMD entry 2992; Maskell *et al.*, 2015 ▸). (*b*) NuA4 acetylase–NCP structure (EMD entry 9536; Xu *et al.*, 2016 ▸). (*c*) 53BP1–NCP-ubme structure (EMD entry 8246; Wilson *et al.*, 2016 ▸). Left inset: magnified view of the UDR-ubiquitin–NCP interaction with ubiquitin fitted into density (PDB entry 1ubi). (*d*) Chp1 chromodomain–NCP-me structure (EMD entry 8063; Zocco *et al.*, 2016 ▸). (*e*) 12-mer chromatin fibre (EMD entry 2600; Song *et al.*, 2014 ▸). The left inset highlights a single nucleosome with key features annotated. The crystal structure of NCP (Davey *et al.*, 2002 ▸; PDB entry 3lz0) is fitted into the cryo-EM density.

More recently, a number of subnanometre-resolution structures of NCP–chromatin-binding protein complexes have been determined (Maskell *et al.*, 2015 ▸; Zocco *et al.*, 2016 ▸; Xu *et al.*, 2016 ▸; Wilson *et al.*, 2016 ▸; Fig. 2 ▸). At local resolutions in the range of <9 Å, secondary-structure elements (α-helices in particular) are more readily resolved, allowing the docking of available atomic coordinates and the reliable positioning of functional domains within a macromolecular assembly. Moreover, the turn of the DNA double helix can be unambiguously observed, providing a tool to establish of the handedness of a cryo-EM map. Thus, these studies have allowed greater insight into more diverse biological processes from NCP modification and recognition to viral DNA integration.

The structure of the prototype foamy virus intasome bound to an NCP revealed that the target-strand capture leading to retroviral integration occurs in the context of an intact nucleosome and explained why NCPs are preferred over naked DNA as integration substrates (Maskell *et al.*, 2015 ▸). Two separate sites on the nucleosomal DNA are recognized by integrase at positions opposite to the NCP dyad. The integration site is stabilized by a number of protein contacts that involve one H2A–H2B dimer (Fig. 2 ▸ *a*). Here, nucleosomal DNA is lifted and underwound to allow access to the integrase catalytic core. Whether nucleosomal DNA is reshaped upon integration remains to be established. A secondary docking site involves an integrase contact with the second gyre in the NCP DNA (Fig. 2 ▸ *a*). Amino-acid substitutions in the NCP-contacting elements of integrase affect both integration efficiency *in vitro* as well as the viral integration landscape in cells (Maskell *et al.*, 2015 ▸).

The budding yeast Tip60–NuA4 complex acetylates H4 to regulate transcription and DNA repair (Doyon & Côté, 2004 ▸). Complementing multiple crystal structures of the truncated four-subunit NuA4 complex, Xu and coworkers determined a cross-linked 7.9 Å resolution structure of the NuA4 core in complex with an NCP (Xu *et al.*, 2016 ▸; Fig. 2 ▸ *b*). The structure revealed how the low-specificity acetylase is confined to modify only lysines in the N-terminal tails of histone H4: the NuA4 complex engages the nucleosome face, orientating the catalytic Esa1 subunit over the H4 tail. To establish this elegant spatial recognition pattern the NuA4 complex forms extensive contacts with the NCP, primarily through a reconfiguration of the Epl1 subunit, binding both dyad-adjacent DNA and the NCP acidic patch. A semi-flexible Tudor domain within Esa1 was docked into density proximal to the cata­lytically engaged Esa HAT domain, in close proximity to nucleosomal DNA. Interestingly, the complete NuA4 complex contains chromatin reader domains that are not present in this study but are required to direct histone acetylation (Steunou *et al.*, 2016 ▸). How these extra domains interact within an NCP remains an unanswered question.

## 4. Studying histone modifications using cryo-EM   

NCPs become decorated with a wide range of post-translational modifications, which directly control DNA accessibility and binding to specific interactors. In turn, these histone-binding factors can alter the structural properties of chromatin, helping to coordinate DNA-related processes in the cell. The available crystal structures focus on isolated domains bound to short stretches of modified peptide. Indeed, it is clear that many proteins exhibit a higher affinity for chromatin than would be expected from a simple binding event to a short linear primary sequence. Numerous studies have now shown the critical relevance of analysing modified chromatin interaction within the context of an NCP (Xu *et al.*, 2016 ▸; Bartke *et al.*, 2010 ▸; Nikolov *et al.*, 2011 ▸; Ng & Cheung, 2016 ▸). This suggests that the common theme of multivalent binding of chromatin proteins to the nucleosome surface also extends to the recognition of post-translationally modified NCPs in the form of the ‘histone code’. The majority of post-translational modifications are found on the disordered histone tails, and little structural information is available on how covalent modifications affect an NCP.

Producing large quantities of modified NCPs is a challenging task, which has impaired rapid advancement in our structural understanding of the histone code. Ubiquitylation has proved to be a tractable modification for the large-scale production required for structural studies (Machida *et al.*, 2016 ▸; Morgan *et al.*, 2016 ▸), thanks in part to advances in biological chemistry (Faggiano & Pastore, 2014 ▸). Morgan and coworkers utilized multiple chemical approaches to produce homogenous quantitates of NCPs containing H2B Lys-120ub (Morgan *et al.*, 2016 ▸). The 3.8 Å resolution crystal structure of the SAGA DUB module bound to NCP-H2BK120ub helped to explain how the multi-subunit DUB is directly targeted to remove this modification in chromatin. One additional challenge is represented by the inherent flexibility of the large ubiquitin protein modification on the substrate, which has prevented complete model building and localization of the ubiquitin modification (Machida *et al.*, 2016 ▸; Morgan *et al.*, 2016 ▸; Wilson *et al.*, 2016 ▸; Li *et al.*, 2017 ▸).

A recent study revealed how multiple post-translational modifications and the surface of an NCP can combine to confer specificity and affinity to a chromatin-binding component. 53BP1 is a key DNA damage-response factor that is implicated in the repair of DNA double-strand breaks (Panier & Boulton, 2014 ▸) and is recruited to DNA damage-adjacent chromatin through the recognition of a methyl mark on the tail of H4 (H4K20me2) and a DNA damage-inducible mark: H2A Lys15 ubiquitylation (H2AK15ub). A short fragment of 53BP1 is sufficient for the recruitment to sites of DNA lesions in cells and comprises an H4K20me2-interacting tandem Tudor domain (Botuyan *et al.*, 2006 ▸) and a short region termed the ubiquitylation-dependent recruitment region (UDR; Fradet-Turcotte *et al.*, 2013 ▸).

The 4.5 Å resolution cryo-EM structure of 53BP1 bound to a methyllysine analogue and ubiquitylated NCP revealed that 53BP1 forms direct contacts with histone-tail methylation and ubiquitylation modifications, as well as the nucleosome surface itself (Wilson *et al.*, 2016 ▸; Fig. 2 ▸ *c*). A chemical approach was employed to create a dimethyllysine analogue on histone H4 Lys20. Tandem Tudor domain binding was limited to just the modified H4 histone tail; the reconstituted map had weaker density tethered over the H4 tail and concurrently poorer resolution, suggesting flexible binding without stable association with the NCP surface. The better ordered peptidic UDR snakes over the NCP surface and sandwiches between the nucleosome and ubiquitin, fixing ubiquitin in a constrained conformation. Ubiquitin recognition was garnered by interactions between the UDR, the histone surface and a constrained ubiquitin, which was folded over the NCP surface. This recognition mode helped to explain the site specificity of 53BP1 for H2AK15ub. Density for the UDR was sufficient to allow model building of this segment, and sequence register was inferred by complementary biochemistry and cross-linking. This revealed that the previously identified key 53BP1 residues (Fradet-Turcotte *et al.*, 2013 ▸) interact directly with ubiquitin, while a basic stretch of residues in the UDR interact with the H2A–H2B acidic patch in a manner resembling other acidic patch-interacting proteins.

Cryo-EM was used to determine how a chromodomain from fission yeast Chp1 reads the heterochromatic H3K9me3 mark (Zocco *et al.*, 2016 ▸; Fig. 2 ▸ *d*). By integrating the crystallographic data for the Chp1 chromodomain (Schalch *et al.*, 2009 ▸) with the 10 Å resolution cryo-structure of Chp1 in complex with a methylated NCP, the Chp1-binding module was located over the core of the NCP, rather than near the H3 N-terminal tail. Based on this assignment, the Chp1 module is poised to contact the acidic patch, the H4 tail and the core of histone H3. The authors suggest that recognition of the H3K9me3 tail would require the tail to loop back towards the NCP core before entry into the Chp1 binding site, orthogonal to the NCP surface.

## 5. Structural flexibility of chromatin complexes analysed by cryo-EM   

In cryo-EM, the rapid freezing of proteins into vitreous ice hopes to recapitulate the status of proteins in solution. Indeed, a diverse set of conformational states of the same macromolecular assembly can be isolated from an EM data set *in silico*. The nominal reported resolution reflects a global estimate derived from the entire three-dimensional structure. Owing to the nature of single-particle averaging in electron microscopy, an EM structure can span a large resolution range, providing high-resolution information on a structured core as well as information on conformational variability at the particle periphery. As a result, in comparing EM and crystallo­graphic structures it should be noted that the methods for estimating resolution are inherently different. The local resolution of EM maps can be calculated by *ResMap* (Kucukelbir *et al.*, 2014 ▸), allowing direct quantitation of the fluctuations in local map resolution. This data can be displayed in the form of heat maps and allows comparison not only within a structure but also between structures of comparable resolution, often providing important mechanistic insights.

This tool has proven useful, for example, to help compare the relative rigidity of ubiquitin attached to NCP in the presence or absence the cognate binding partner 53BP1 (Wilson *et al.*, 2016 ▸). This analysis revealed that the ubiquitin attached to the NCP was highly motile, tethered only *via* its covalent attachment to the tail of histone H2A. When 53BP1 was bound to the modified NCP complex, however, a clear ordering of the covalently attached ubiquitin appeared evident (Fig. 2 ▸ *c*).

Analysis of the local resolution can be used to describe the flexibility of nucleosomal DNA. In recently determined structures the DNA is at a slightly poorer resolution compared with the histones, suggesting that the DNA displays a small degree of flexibility (Chua *et al.*, 2016 ▸; Wilson *et al.*, 2016 ▸).

## 6. Using cryo-EM to image higher order chromatin structures   

Early rotary shadowing EM studies of partially unfolded chromatin revealed a characteristic ‘beads-on-a-string pattern’ of regularly spaced NCP arrays connected by linker DNA (Thoma & Koller, 1977 ▸). How more than 2 m of DNA is further compacted in the nucleus of each human cell has been the subject of intense research efforts. Higher order chromatin is likely to be arranged in multiple mixed states (Kuznetsova & Sheval, 2016 ▸). Cryo-EM has helped to reveal how one model of chromatin compaction, the ‘30 nm fibre’, may occur. 30 nm-like structures can be formed using *in vitro* reconstituted nucleosome arrays incubated with linker histone (Song *et al.*, 2014 ▸), similar to those observed in cells (Scheffer *et al.*, 2011 ▸; Li *et al.*, 2015 ▸; Finch & Klug, 1976 ▸).

Song and coworkers visualized cross-linked fibrils of 12 positioned NCPs formed by adding linker histone H1.4 (Song *et al.*, 2014 ▸; Fig. 2 ▸ *e*). The structure (solved to 11 Å resolution) revealed that the fibril forms a left-handed double helix with a zigzag pattern of NCPs bridged by linker DNA. This architecture of the 30 nm fibre agrees well with the ‘two-start model’ suggested by the tetranucleosome crystal structure, whereby straight linker DNA bridges between two adjacent stacks of NCPs (Schalch *et al.*, 2005 ▸). Interestingly, the fibre did not form a contiguous helix. Instead, tetranucleosomal units stack on top of each other with changes in pitch to create the helical pattern. Multiple intra-NCP interactions are visible; H2A–H2B four-helical bundles are formed from neighbouring NCPs within a tetrameric unit. Between tetramer units a much larger gap is bridged by the tail of one histone H4 projecting into the H2A–H2B acidic patch of a neighbouring NCP. Notably, this arrangement recapitulates an interaction seen in the crystal packing of single NCP structures (Luger *et al.*, 1997 ▸). Density for histone H1.4 was placed near the dyad of every NCP in the fibril; asymmetrically placed near the DNA dyad to interact with both exit and entry DNA. This asymmetry confers polarity to the fibre, and H1–H1 dimeric interactions between nucleosomes stabilize the tetranucleosome units. Changing the length of the linker DNA alters both the diameter and height of the fibre without affecting the overall stacking in the structure. Interestingly, the subsequent structure of an alternate linker histone, histone H5, bound to an NCP (Zhou *et al.*, 2015 ▸) could help to explain a different observed topology of chromatin-fibre folding (Robinson *et al.*, 2006 ▸). H5 binds symmetrically to the NCP, leading to a different trajectory of linker DNA and possibly altering the fibre architecture.

## 7. Future perspectives   

We currently lack a molecular understanding of how most chromatin-binding proteins interact with nucleosomal DNA, making the study of chromatin superstructure an exciting emerging field. Cryo-EM is an important addition to the structural biologist’s toolkit and will enable us to visualize increasingly complex biological systems centred on chromatin. Indeed, as we have outlined, cryo-EM offers a unique tool to help to investigate previously intractable nucleosome-bound factors. Unlike other structural biology techniques, the visualization of macromolecules in cryo-EM is only limited by their biochemical formation, their stability and the ability to discern particle orientations in the micrographs. Nevertheless, optimizing grid freezing and imaging conditions in cryo-EM is still a laborious task that prevents high-throughput structure determination at present.

Despite the significant technical advances in cryo-EM, an atomic resolution structure of frozen hydrated NCPs is still to be achieved. To date, NCP-bound EM structures have used hybrid methods that combine docking high-resolution fragments into a high-order structure to interpret the cryo-EM density. This approach has allowed inferences at the residue level, which can be further validated in a biochemical or cell-biological setting. Model building of a peptide backbone and secondary-structure features can be performed at resolutions in the range of 4 Å; however, this model still requires extensive downstream validation in order to ensure that the correct sequence register is achieved. With the advent of faster image processing, more affordable access to high-end microscopes and renewed interest in the technique, we are likely to enter a golden age of molecular understanding of chromatin biology.
